# Neurological complications after cardiac surgery: a retrospective case-control study of risk factors and outcome

**DOI:** 10.1186/s13019-019-0844-8

**Published:** 2019-01-25

**Authors:** Giuseppe Maria Raffa, Francesco Agnello, Giovanna Occhipinti, Roberto Miraglia, Vincenzina Lo Re, Gianluca Marrone, Fabio Tuzzolino, Antonio Arcadipane, Michele Pilato, Angelo Luca

**Affiliations:** 1 Cardiac Surgery and Heart Transplantation Unit, Department for the Treatment and Study of Cardiothoracic Diseases and Cardiothoracic Transplantation, IRCCS – ISMETT (Istituto Mediterraneo per i Trapianti e Terapie ad alta specializzazione), Via Tricomi 5, 90127 Palermo, Italy; 2 Diagnostic and Therapeutic Services, Radiology Unit, IRCCS – ISMETT (Istituto Mediterraneo per i Trapianti e Terapie ad alta specializzazione), via Tricomi 5, Palermo, 90127 Italy; 3 Department of Anesthesia and Critical Care, IRCCS – ISMETT (Istituto Mediterraneo per i Trapianti e Terapie ad alta specializzazione), via Tricomi 5, Palermo, 90127 Italy; 4 Neurology Service, Department of Diagnostic and Therapeutic Services, IRCCS – ISMETT, via Tricomi 5, Palermo, 90127 Italy; 5 Statistician, Research Office, IRCCS ISMETT (Mediterranean Institute for Transplantation and Advanced Specialized Therapies), Via Tricomi 5, 90127 Palermo, Italy

**Keywords:** Atherosclerosis, Carotid arteries, CT-scan, MRI, Neurocognitive deficits, Cardiac surgery

## Abstract

**Background:**

To evaluate incidence, risk factors, and outcomes of postoperative neurological complications in patients undergoing cardiac surgery.

**Methods:**

A total of 2121 patients underwent cardiac surgery between August, 2008 and December, 2013; 91/2121 (4.3%) underwent brain computed tomography (70/91, 77%) or magnetic resonance imaging (21/91, 23%) scan because of major stroke (37/2121, 1.7%) and a spectrum of transient neurological episodes as well as transient ischemic attacks and delirium /psychosis/seizures (54/2121, 2.5%). The mean age was 65.3 ± 12.1 years and 60 (65.9%) were male. Variables were compared among study- and matched-patients (*n* = 113) without neurological deficits.

**Results:**

A total of 37/2121 (1.7%) patients had imaging evidence of stroke. Radiological examinations were done 5.72 ± 3.6 days after surgery. Patients with and without imaging evidence of stroke had longer intensive care unit length of stay (LOS) (13.8 ± 14.7 and 12.9 ± 15 days vs. 5.7 ± 12.1 days, respectively (*p* < 0.001) and hospital LOS (53 ± 72.8 and 35.5 ± 29.8 days vs. 18.4 ± 29.2 days, respectively (p < 0.001) than the control group. The hospital mortality of patients with and without imaging evidence of stroke was higher than the control group (7/37 patients [19%], and 12/54 patients [22%] vs. 4/115 patients [3%], respectively (p < 0.001). Multivariate analysis showed that bilateral internal carotid artery stenosis of any grade (*p* < .001), and re-do operations (*p* = .013) increased the risk of postoperative neurological complications.

**Conclusions:**

Neurological complications after cardiac surgery increase hospitalization and mortality even in patients without radiologic evidence of stroke. Bilateral internal carotid artery stenosis of any grade, suggesting a diffuse patient propensity toward atherosclerosis, and re-do operations increase the risk of postoperative neurological complications.

## Background

Postoperative neurological impairment after cardiac surgery is a serious complication [1–3]. Clinical manifestations are various, and include cognitive-behavioral disturbances, altered states of consciousness, and focal neurological deficits [4–8]. Neurocognitive impairment occurs in 15–66% of patients at discharge, and in up to 40% of patients at 5 years after surgery [9, 10]. Symptomatic stroke occurs in 1.2 to 6% of patients, and its incidence is higher in the elderly [11–14]. Several studies have evaluated postoperative neurologic complications and risk factors in cardiac surgery patients, though the results are conflicting, or incomplete mainly due to a low number of interventions or a low number of variables. Some studies have reported the association between prolonged cardiopulmonary bypass and the development of postoperative stroke [11, 14], while others have questioned its significance [11, 13]. Few studies have evaluated the incidence of postoperative neurological complications in a large group of patients who have undergone a variety of cardiac procedures [10, 12, 14].

Therefore, the purpose of our study was to identify the incidence of neurological complications and their risk factors in patients who underwent cardiac surgery at our institute.

## Methods

### Study design

This retrospective study was carried out in a major referral hospital for cardiovascular diseases [8, 15–19]. Our Institutional Research Review Board approved this study, with a waiver of informed consent. Between August, 2008 and December, 2013, 2121 patients underwent cardiac operations at our institute. Among them, 91/2121 (4.3%) patients, with no history of previous cerebrovascular events, suffered postoperative neurological complications and underwent either computed tomography (CT) or magnetic resonance imaging (MRI) brain imaging. Postoperative neurologic complications were defined as any newly developed central neurological deficit occurring before the discharge of the patient. The severity of neurological complications was classified into major strokes (associated with permanent major disabilities and evident lesion on neurological imaging) and transient ischemic attacks and delirium/psychosis/convulsion (reversible neurological symptoms with no significant lesions on neurological imaging). A further classification according to the size of neurological injury detected on CT or MRI was made (see brain imaging protocol and analysis section). A control group was matched in order to identify predictors of neurological complications. The control group was composed of 113 cardiac surgery patients without neurological complications selected according to age, gender, and type of intervention. For each patient, intensive care unit length of stay (ICU-LOS) and overall hospital LOS were recorded.

### Surgical procedures

Surgical procedures were performed by four faculty level cardiovascular surgeons. Surgery consisted of 1) coronary artery bypass graft (CABG) (*n* = 54); 2) valve operations (*n* = 40) [15, 17]; 3) complex procedures (CABG and aortic surgery, combined aortic, mitral, tricuspid valve surgery, heart transplantation [16], left ventricle assist device [18], left ventricular aneurysmectomy, repair of post-infarction ventricular septal defect) (*n* = 44); 4) aortic procedures (*n* = 49) [19]; and 5) re-do operations (*n* = 17). Details are listed in Table 1. Classification was made according to the different thromboembolic risks, de-airing issues, and brain protection strategies.

**Table 1:** Patient characteristics and analysis of continuous (mean and ranges) and categorical variables (%)

Variable	Study group (*n* = 91)	Matched patients (*n* = 113)	*P* value
*Preoperative characteristics*			
Age (years)	65.4 (25–68)	65.4 (29–84)	0.99
Gender (M)	60 (65%)	73 (64%)	0.13
Height (cm)	163 (147–184)	163 (143–192)	0.64
Weight (kg)	72.31 (48.8–114)	74 (44.2–135.5)	0.39
BMI	27.07 (17.5–41.7)	27.5 (16–43.06)	0.52
BSA	1.8 (1.43–2.32)	1.82 (1.35–2.58)	0.35
Diabetes	32 (35%)	31 (27%)	0.36
Hypertension	66 (72%)	85 (75%)	0.64
Dyslipidemia	34 (37%)	45 (40%)	0.66
Chronic Renal Failure	10 (11%)	12 (10%)	1
Smoking			0.94
Nonsmoker	49 (54%)	55 (49%)	
Current smoker	15 (16%)	25 (22%)	
Former smoker	27 (30%)	33 (29%)	
Family history of stroke	19 (21%)	29 (26%)	0.52
Bilateral ICA stenosis < 50%	52 (57%)	9 (8%)	*<.001*
Bilateral ICA stenosis of any grade	63 (69%)	17 (15%)	*<.001*
*Surgical Procedure*			
CABG	25/91 (27%)	29/113 (26%)	0.77
Valve operations	14/91 (15%)	26/113 (23%)	0.17
Aortic valve repair/replacement	5	12	
Mitral valve repair/replacement	9	14	
Complex procedures	16/91 (18%)	28/113 (24%)	0.21
CABG + Aortic surgery	4	7	
Combined aortic, mitral and tricuspid valve	4	9	
Heart transplantation	3	7	
LVAD	3	3	
Left ventricular aneurysmectomy	1	1	
Repair of post-infarction VSD	1	1	
Aortic procedures	24/91 (27%)	25/113 (22%)	0.48
Ascending aorta surgery	5	7	
Aortic arch surgery	2	5	
Combined procedure	10	7	
Aortic root surgery	3	3	
Valve operation + Aortic procedure	3	1	
CABG + Aortic procedure	1	2	
Re-do Operation	12/91 (13%)	5/113 (5%)	*0.02*
*Surgical Details*			
CBP time (minutes)	111.11 (0–257)	99.12 (0–257)	0.10
Aortic clamp time (minutes)	77.67 (0–216)	71.47 (0–197)	0.28
Hypothermic circulatory arrest	25 (27%)	17 (15%)	*0.02*
On-pump CABG with beating heart	7 (7%)	3 (3%)	0.10
Off-pump CABG	4 (4%)	6 (5%)	0.2
Number of distal anastomoses (from 1 to 5)	40 (44%)	44 (39%)	0.3
Intraoperative hemofiltration	3 (3%)	2 (2%)	0.07
Complicated weaning from CPB	12 (13%)	5 (4%)	*0.02*
Cardioplegia delivery			
Anterograde	70 (77%)	86 (76%)	0.12
None	11 (12%)	9 (8%)	
Anterograde+selective	7 (8%)	12 (11%)	
Anterograde+ retrograde	3 (3%)	6 (5%)	
Cardioplegia carrier			
Crystalloid	8 (10%)	3 (3%)	*0.02*
Blood	72 (90%)	110 (97%)	
Cardiac rhythm at sternal closure			
Sinus	37 (41%)	61 (54%)	*0.04*
Non sinus (AF, AVB, PM)	54 (59%)	52 (46%)	
Defibrillation required at weaning	23 (25%)	23 (20%)	0.31
Surgery time (minutes)	152.24 (21–368)	132.08 (35–660)	0.07
Intra-aortic balloon pump	9 (10%)	9 (8%)	0.62
*Post-operative data*			
Severe hypotension	20 (22%)	25 (22%)	0.86
Need for vasoactive drugs	55 (60%)	54 (48%)	*0.03*
Transfusion of red blood cells (units)	2 (2–15)	1.6 (0–22)	0.26
Transfusion of plasma (units)	1.75 (0–18)	1.34 (0–27)	0.32
Transfusion of platelets (units)	3.1 (0–22)	2.34 (0–20)	0.21

*BMI* body mass index, *BSA* body surface area, *ICA* internal carotid artery, *CABG* coronary artery bypass graft, *LVAD* left ventricle assist device, *VSD* ventricular septal defect, *CPB* cardiopulmonary bypass, *AF* atrial fibrillation, *AVB* atrio-ventricular block, *PM* pacemaker

### Risk factors

Clinical and surgical charts were reviewed in order to identify risk factors associated with postoperative stroke. Preoperative variables included degree of internal carotid artery (ICA) stenosis on color Doppler ultrasound (US); height; weight; body mass index; body surface index; arterial hypertension; diabetes; dyslipidemia; chronic renal failure (creatinine > 1.8 mg/dl); and smoking status (nonsmoker, current smoker, or former smoker). Operative variables included use of cardiopulmonary bypass (CPB); cardiac rhythm at sternal closure; surgery time; need for intra-aortic balloon pump; number of anastomoses; complicated weaning from CPB; need for vasoactive drugs; severe hypotension (mean arterial pressure < 50 mmHg) during surgery; and transfusion of packed red blood cells, platelets, or fresh frozen plasma. When cardiopulmonary bypass was used, cardiopulmonary bypass time, aortic clamp time, hypothermic circulatory arrest of any grade ranging from 24 °C to 28 °C, cardioplegia delivery (anterograde or retrograde), and carrier (crystalloid or blood), and intraoperative hemofiltration were recorded.

### ICA Doppler US and analysis

ICA Doppler US was done as part of preoperative imaging work-up within three months before cardiac surgery. Doppler US was done by experienced radiologists using Logiq7 or Logiq9 technology (GE Medical Systems, Milwaukee, USA) and a 9 MHz sound linear transducer. Doppler images were retrospectively reviewed, and the degree of ICA stenosis was classified according to the Society of Radiologists in Ultrasound Consensus Conference criteria [20]. ICA stenosis was graded as absent (peak Systolic Velocity (PSV) less than 125 cm/sec and no visible plaque or intima-media thickness) or present with < 50% stenosis (PSV less than 125 cm/sec and visible plaque or intimal thickening); 50–69% stenosis (PSV equal to 125–230 cm/sec and visible plaque); 70% stenosis to near occlusion (PSV greater than 230 cm/sec and visible plaque and luminal narrowing); near occlusion (evidence of a markedly narrowed lumen on color Doppler US and not applicable velocity parameters); and total occlusion (no sonographically detectable patent lumen and no flow on color Doppler US) (Table 2). ICA stenosis side was also classified as right, left, or bilateral.

**Table 2:** Internal carotid artery ultrasound findings

ICA	Population	Normal	< 50%		50–70%	> 70% to near occlusion	Occlusion
*Right*	*Study (n = 91)*	14 (15.4%)	70 (77%)		3 (3.3%)	3 (3.3%)	1 (1%)
	*Matched (n = 113)*	90 (79.7%)	17 (15%)		2 (1.8%)	3 (2.6%)	1 (0.9%)
*Left*	*Study (n = 91)*	15 (16.5%)	72 (79.2%)		–	3 (3.3%)	1 (1%)
	*Matched (n = 113)*	95 (84%)	16 (14.2%)		1 (0.9%)	1 (0.9%)	

*ICA* Internal carotid artery

### Brain imaging protocol and analysis

MRI was done on a 1.5 Tesla scan (Signa HDXT, GE Medical Systems, Milwaukee, USA). MRI protocol included an axial T2-weighted fluid attenuation inversion recovery (FLAIR) sequence; an axial T2-weighted fast spin echo sequence; an axial T2-weighted gradient echo sequence; an axial diffusion-weighted sequence using b values of 0 and 1000 s/mm^2^; a sagittal T1-weighted spin echo sequence; a three-dimensional T1-weighted spoiled gradient echo sequence (contrast dye was not routinely used). CT was done on a 64-section CT scan (VCT 64; GE Medical Systems, Milwaukee, Wis) and, again, contrast dye was not routinely used. Patients with stroke underwent emergent CT or MRI as soon as symptoms occurred and the diagnostic tool used were repeated 24 h after symptoms or whenever clinical changes occurred. In patients received brain CT scan, the 24 h re-evaluation allowed the detection of the vascular territory involved corresponding to the clinical neurological features. Brain images were independently evaluated by two experienced radiologists. Both readers were blinded to clinical information. Diagnosis of stroke was made using previously published criteria [21, 22]. Strokes were classified into ischemic and hemorrhagic. Ischemic strokes were further classified into the following categories: 1) small stroke; 2) not small stroke; 3) coexisting small and not small strokes. Small strokes were less than 5 mm in diameter. Not small strokes included strokes greater than 5 mm in diameter. Stroke arterial distribution and number were recorded.

### Statistical analysis

Quantitative variables are expressed as mean ± standard deviation, and qualitative variables as absolute and relative frequencies. Quantitative variables were compared using the paired t-test, the Wilcoxon-Mann-Whitney test, or analysis of variance (ANOVA) when were appropriate. Qualitative variables were compared using Fisher’s exact test. Preoperative and operative variables were compared among patients with neurological complications (study group) and those without (control group). ICA stenosis was further classified as bilateral and not bilateral. Bilateral ICA stenosis included ICA stenosis of any degree. A multiple logistic regression analysis was used to assess the independence of the risk factors of neurological complications. The multiple logistic regression model included variables with *p* values ≤0.2 from univariate analysis adjusted by type of intervention. A significance level of 0.2 was used to remove variables from the model by stepwise selection.

Outcome analysis evaluated the ICU-LOS, the overall hospital LOS and the hospital mortality. Outcome variables were compared among neurological patients with imaging evidence of stroke and control group patients, among neurologic patients without imaging evidence of stroke and control group patients, and among neurological patients with and without imaging evidence of stroke. A *p*-value of <0.05 was considered statistically significant. Statistical analysis were done with STATA, version 13.1software (StataCorp LP, College Station, Texas, USA).

## Results

### Patient characteristics

Among the 91 study group, 37/2121 (1.7%) had major strokes and 54/2121 (2.5%) had transient ischemic attacks (including delirium/psychosis/convulsion). The mean age was 65.3 ± 12.1 years, and 60 (65.9%) were male.

### Imaging findings

**Fig. 1:**
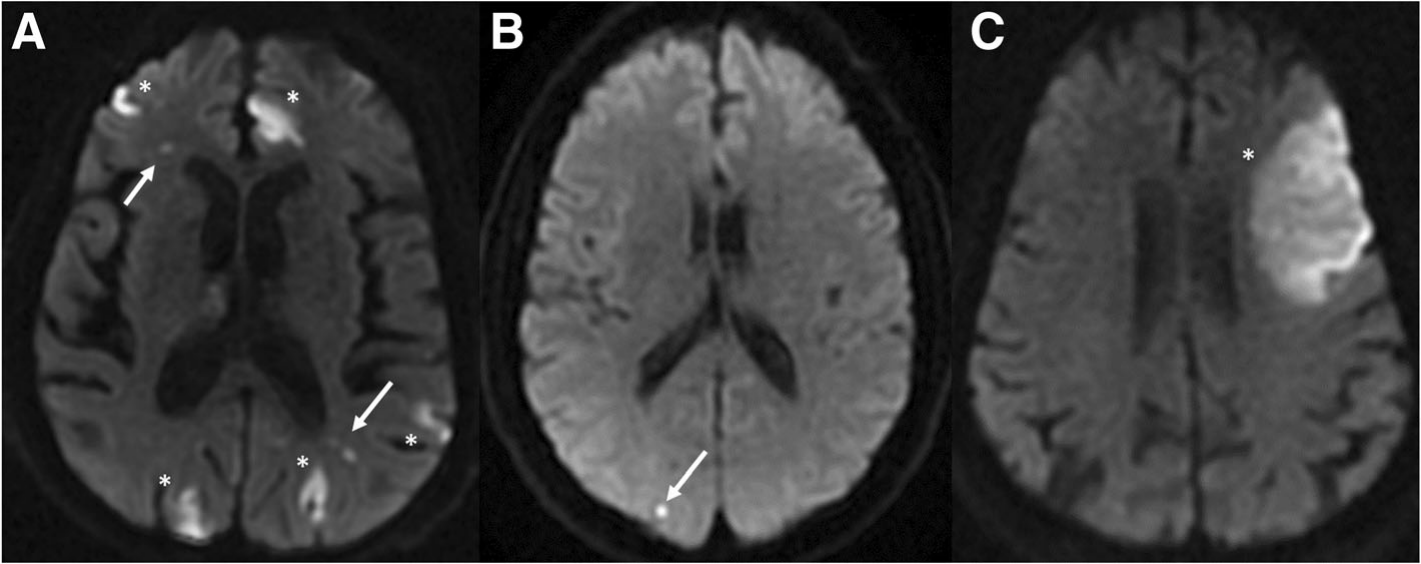
**a** Axial DWI obtained in a 69-year-old man two days after cardiac surgery shows multiple small (arrow) and not small (*) ischemic strokes in both hemispheres. The patient had bilateral < 50% internal carotid artery (ICA) stenosis. Absence of significant ICA stenosis and bilateral distribution suggest an embolic origin of the strokes. **b** Axial DWI obtained in a 72 year-old woman two days after cardiac surgery shows a small hyperintense area (arrow) in the right posterior cerebral artery territory, consistent with small ischemic stroke. The patient had bilateral < 50% ICA stenosis. **c** Axial DWI obtained in a 69-year-old man three days after cardiac surgery shows a large, hyperintense area in the left middle cerebral artery territory, consistent with not small ischemic stroke. The patient had bilateral < 50% ICA stenosis

Radiological examinations were done 5.72 ± 3.6 days after surgery. Brain imaging was performed using CT in 70/91 (77%) and MRI in 21/91 (23%) patients. Thirty-seven/91 (41%) patients had imaging evidence of stroke and 35/37 (94%) had ischemic etiology (25/35 were not-small strokes, 6/35 were small strokes; and 4/35 were both small and not small strokes, respectively) (Fig. 1, a-c). Ischemic stroke was single in 21/35 (60%) patients and multiple in 14/35 (40%) patients. Ischemic strokes were located as follows: middle cerebral artery territory in 10/35 (28%) patients; posterior cerebral artery territory in 15/35 (43%) patients; posterior inferior cerebellar artery territory in 1/35 (3%); and more than 1 arterial territory in 9/35 (26%) patients. Two/37 (6%) patients had hemorrhagic strokes. All hemorrhagic strokes (2/37, 6%) were single, and were located in the left middle cerebral artery territory, in the area of the deep perforating lenticulostriate arteries.

### Risk factors

Univariate analysis showed that bilateral ICA stenosis of any grade, hypothermic circulatory arrest, crystalloid cardioplegia, lack of sinus rhythm at sternal closure, difficult weaning from CPB, and vasoactive drugs were associated with postoperative neurological complications. Other preoperative and operative variables were not statistically different among groups. Details are shown in Table 1.

Variables in the multivariate analysis included bilateral ICA stenosis of any grade; off-pump CABG; on-pump CABG with beating heart; hypothermic circulatory arrest; complicated weaning from CPB; use of crystalloid cardioplegia; vasoactive drugs; and type of surgery. Multivariate analysis showed that bilateral ICA stenosis of any grade (odds ratio: 73.84; 95% confidence interval, 21.87–249.29; *p* < .001), and re-do operations (odds ratio: 7.99; 95% confidence interval, 1.58–51.24; *p* = .013) increased the risk of neurological complications.

### Outcomes

**Fig. 2:**
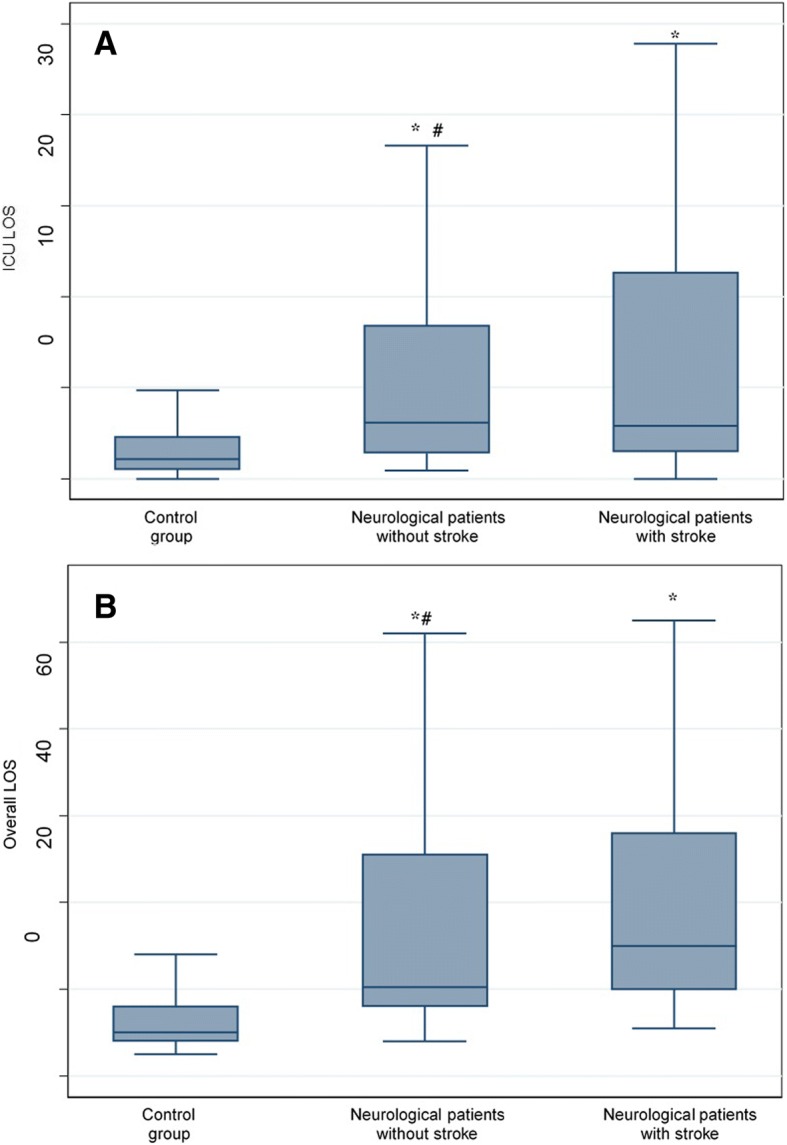
Box-and-whisker plot of intensive care unit length of stay (LOS) (**a,** *p < .001 compared with control group, ^#^*p* = .065 compared with neurological patients with imaging evidence of stroke**)** and hospital LOS (**b,** *p < .001 compared with control group. ^#^*p* = .073 compared with neurological patients with imaging evidence of stroke). Horizontal line indicates median. Upper and lower margins of the box indicate 75th and 25th percentile of values, respectively. Whiskers indicate ranges

**Fig. 3:**
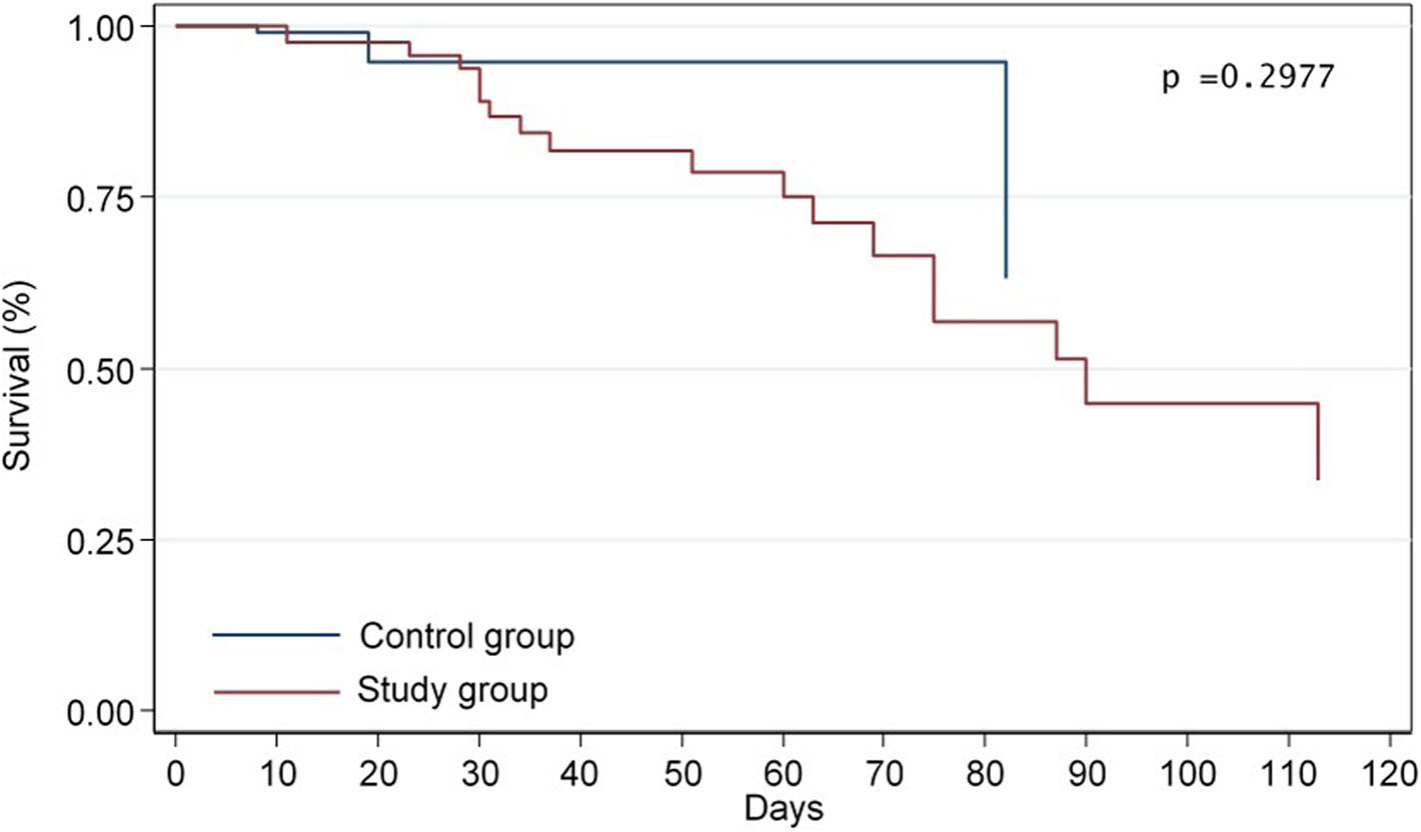
Kaplan Meyer Curve for hospital mortality in control group and study group

Patients with and without imaging evidence of stroke had longer ICU-LOS (13.8 ± 14.7 and 12.9 ± 15 days vs. 5.7 ± 12.1 days, respectively (*p* < 0. 001) and hospital LOS (53 ± 72.8 and 35.5 ± 29.8 days vs. 18.4 ± 29.2 days, respectively (*p* < 0.001) than the control group. The hospital mortality of patients with and without imaging evidence of stroke was higher than the control group (7/37 patients [19%] and 12/54 patients (22%) vs. 4/115 patients (3%), respectively (*p* < 0.001) (Fig. 2a, b). However, Kaplan Meier analysis showed no statistically significant difference in temporal occurrence of hospital mortality among the study group and control group (*p* = 0.2977) (Fig. 3). There was no statistically significant difference in outcome variables between neurological patients with imaging evidence of stroke and those without (Fig. 2 a, b).

## Discussion

In this study we evaluated the incidence of postoperative neurological complications in 2121 consecutive cardiac surgery patients, and found that 4.3% patients had neurological deficits. Thirty-seven/2121 (1.7%) patients had imaging evidence of stroke, and up to 71% showed not-small ischemic strokes. The occurrence of postoperative neurological complications increased hospitalization and hospital mortality. Therefore, it is crucial to identify potential risk factors to prevent postoperative neurological complications. The etiology of postoperative stroke in cardiac surgery patients is multifactorial, with embolism as the predominant cause, while intraoperative hypotension and hemorrhage are less frequent [23]. Embolism is attributed mainly to manipulation of the atherosclerotic aorta, release of gaseous or particulate matter from the cardiopulmonary bypass pump, postoperative atrial fibrillation and minimally invasive procedures [2, 3, 23–25]. Independent risk factors on multivariate analysis associated with neurologic damage were bilateral ICA stenosis and re-do operations.

### Bilateral ICA stenosis

Bilateral ICA stenosis of any grade was the most important risk factor, with an odds ratio of 73.84. Studies have evaluated the relationship between stroke after cardiac surgery and carotid stenosis [26, 27] that includes two major mechanisms: artery to artery (thrombosis from proximal segment to distal segment) or hypoperfusion (causing border zone infarcts - watershed infarction). Hirotani et al. [26] analyzed 476 patients undergoing CABG, and reported the severity of carotid stenosis as the only independent risk factor of postoperative stroke. To the best of our knowledge, no studies correlate ICA stenosis regardless of degree and neurologic damage. In our series, 69% of patients with neurological complications had bilateral ICA stenosis of any grade, even less than 50% (Tables 1 and 2), while 85% of patients without neurological complications had normal ICAs (Tables 1 and 2). We hypothesized that, regardless of the degree, ICA stenosis may be a marker of systemic atherosclerosis. Indeed, the relationship between the calcifications of the aortic arch and the coronary artery disease has been evaluated [28]. In asymptomatic patients, the carotid intima media thickness [29, 30], and not the severity of carotid stenosis [31], may estimate the risks of stroke and ischemic coronary events. Bilateral ICA stenosis is an important finding, and suggests diffuse atherosclerosis damage [32] in fact, the size and location of brain lesions in our series suggest an embolic mechanism from manipulation of ascending aortic and aortic arch [28]. It can also identify patients who may benefit from other diagnostic tests (e.g., chest CT scan without contrast dye) to detect any ascending aorta calcifications and dangerous source of embolic debridement [28, 33–36].

The optimal staged or combined management of patients requiring carotid endarterectomy and cardiac surgery is a matter of debate [37]. Our strategy includes the treatment of unilateral critical stenosis in symptomatic patients, and bilateral stenosis with a unilateral complete occlusion in the asymptomatic one before cardiac operations. Either percutaneous or surgical treatment is decided on an individual basis.

### Re-do operations

Our study also identifies re-do operations as an independent risk factor for postoperative neurological complications. Re-do operations are more technically demanding than first-time operations. Difficulties are related to sternal re-entry, pericardial adhesions, and management of atherosclerotic vein grafts [7, 38–40]. Moreover, reoperation on aortic and mitral valve carries a high risk of calcium, thrombus, muscle fragments, and valve prosthesis debris embolization [41].

In our study, more than 50% of neurological patients had no stroke (either ischemic or hemorrhagic) imaging findings, and this can in part be explained by our study limitations. Neurological complications can be detect using either CT or MRI. CT and MRI are modalities that can be used with confidence, both having their strengths and weaknesses. The choice is based mostly on the available infrastructure and staff. CT is the imaging available in most hospitals. The recent technical advancements in CT and its speed confer additional advantages to this technique. Radiation exposure remains a relevant issue in stroke CT. MRI can provide additional information and is more sensitive to small ischemic lesions, on the other hand CT is more widely available. Some institutions have MRI available at all times and prefer it over CT for many patients due to the additional information it provides and more sensitivity. Unenhanced brain CT scan has been shown to be relatively non-sensitive in detecting acute and small strokes [42]. MRI is not feasible in all patients because of the presence of metallic implants or pacemakers. However, both CT scan and MRI showed appropriate sensitivity in detecting any brain lesions whenever clinical major strokes occurred.

Similar to neurological patients with imaging evidence of stroke, patients without had longer hospitalization and higher hospital mortality compared with the control group. However, there was no statistically significant difference in outcome among patients with and without imaging evidence of stroke. These observations suggest that the occurrence of postoperative neurological complications worsened the postoperative outcome independent of the evidence of stroke on imaging, and this may be related to both the clinical impairment and the occurrence of further postoperative complications.

### Limitations

First, our study was retrospective and therefore not all patients underwent MRI. Although possibly related to different causes, patients with delirium/psychosis/convulsion and transient ischemic attacks were grouped according to the reversibility of the neurological damage. Patient characteristics before and after matching are missing. Selection of patients was limited to those who had postoperative brain imaging within 15 days; this type of inclusion may induce substantial bias, and also is limited in that there is no good evidence regarding numbers of patients imaged at other institutions for stroke. Second, no follow-up data were available because our protocol requires only one visit, 30 days after discharge. Several variables, including the type of stroke (ischemic or hemorrhagic), the detailed degree of ICA stenosis, use of statins and other medications, the operator, and the length of surgery were not modeled in the analysis. Finally, the study of intracranial arterial system and cerebrovascular tree both at MRI and CT was not performed missing further information of possible causes of stroke.

## Conclusion

This study showed that the occurrence of postoperative neurological complications increases hospitalization and hospital mortality. Among the preoperative and operative variables analyzed, only bilateral ICA stenosis of any grade and re-do operations increased the risk of postoperative neurological complications. Preoperative ICA ultrasound is useful in identifying patients at higher risk of postoperative neurological complications.

## Abbreviation

CABG: coronary artery bypass graft
CPB: cardiopulmonary bypass
CT: computed tomography
ICA: internal carotid artery
ICU-LOS: intensive care unit length of stay
MRI: magnetic resonance imaging
PSV: peak systolic velocity
US: ultrasound
